# Cannabidiol Disrupts Mitochondrial Respiration and Metabolism and Dysregulates Trophoblast Cell Differentiation

**DOI:** 10.3390/cells13060486

**Published:** 2024-03-11

**Authors:** Tina Podinic, Louise Limoges, Cristina Monaco, Andie MacAndrew, Mahek Minhas, Joshua Nederveen, Sandeep Raha

**Affiliations:** 1 Graduate Program in Medical Sciences, Department of Pediatrics, McMaster University, 1280 Main St. W., Hamilton, ON L8S 4K1, Canada; podinict@mcmaster.ca (T.P.); louise.limoges.ll@gmail.com (L.L.); monacc1@mcmaster.ca (C.M.); macandra@mcmaster.ca (A.M.); 2 Department of Pediatrics, McMaster University, 1280 Main St. W., Hamilton, ON L8S 4K1, Canada; minham6@mcmaster.ca (M.M.); nedervj@mcmaster.ca (J.N.); 2 Department of Kinesiology, McMaster University, 1280 Main St. W., Hamilton, ON L8S 4K1, Canada

**Keywords:** cannabis, mitochondria, endocannabinoid system, oxidative stress, differentiation, trophoblast, cytotrophoblast, syncytiotrophoblast, placentation, pregnancy

## Abstract

Trophoblast differentiation is a crucial process in the formation of the placenta where cytotrophoblasts (CTs) differentiate and fuse to form the syncytiotrophoblast (ST). The bioactive components of cannabis, such as Δ^9^-THC, are known to disrupt trophoblast differentiation and fusion, as well as mitochondrial dynamics and respiration. However, less is known about the impact of cannabidiol (CBD) on trophoblast differentiation. Due to the central role of mitochondria in stem cell differentiation, we evaluated the impact of CBD on trophoblast mitochondrial function and differentiation. Using BeWo b30 cells, we observed decreased levels of mRNA for markers of syncytialization (*GCM1, ERVW1, hCG*) following 20 µM CBD treatment during differentiation. In CTs, CBD elevated transcript levels for the mitochondrial and cellular stress markers *HSP60* and *HSP70*, respectively. Furthermore, CBD treatment also increased the lipid peroxidation and oxidative damage marker 4-hydroxynonenal. Mitochondrial membrane potential, basal respiration and ATP production were diminished with the 20 µM CBD treatment in both sub-lineages. mRNA levels for endocannabinoid system (ECS) components (*FAAH, NAPEPLD, TRPV1, CB1, CB2, PPARγ*) were altered differentially by CBD in CTs and STs. Overall, we demonstrate that CBD impairs trophoblast differentiation and fusion, as well as mitochondrial bioenergetics and redox homeostasis.

## 1. Introduction

Cannabis has emerged as an attractive candidate for relief from common symptoms of pregnancy, such as morning sickness. Studies suggest that 2–20% of pregnant people have reported prenatal cannabis use, with 16.2% and 18.1% of pregnant people reporting daily use or matching criteria for abuse and dependence, respectively [1,2,3,4]. Indeed, cannabis use in pregnancy is correlated to adverse pregnancy outcomes, such as stillbirth, intrauterine growth restriction (IUGR) and miscarriages, and neurodevelopmental insufficiencies in adolescence [5,6,7].

Cannabis plants contain over 540 compounds, with Δ9-tetrahydrocannabinol (Δ^9^-THC) and cannabidiol (CBD) being the main bioactive constituents, collectively referred to as phytocannabinoids, that exert either psychoactive or anti-emetic properties [8]. Δ^9^-THC and CBD mainly interact with cannabinoid receptors 1 (CB1) and 2 (CB2) and, in some cases, TRPV1, PPAR**γ** and GPR55 [8]. This endogenous system of cannabinoids, including their receptors and enzymes, is referred to as the endocannabinoid system (ECS) and is highly integrated and characterized in the brain, immune and reproductive tissues [9,10,11,12,13,14]. However, all of the aforementioned cannabinoid-associated receptors as well as two major endocannabinoids, anandamide (AEA) and 2-arachidonoylglycerol (2-AG), are also widely distributed throughout uterine tissue and the placenta [15].

Villous cytotrophoblast cells (CTs) proliferate and fuse, giving rise to syncytiotrophoblasts (STs), which comprise the maternal–fetal interface and provide hormonal support to the developing fetus [16,17,18]. This process is critical not only during early placentation but also during pregnancy to maintain placental function. The placenta is a mitochondria-rich organ as there are heavy energetic demands on this critical organ during pregnancy. Previously, we have demonstrated that 20 µM Δ^9^-THC induces oxidative stress, diminishes mitochondrial respiration, decreases mitochondrial membrane potential and promotes mitochondrial fragmentation in differentiated BeWo cells [19,20]. Although the impacts of CBD on trophoblast mitochondria remain largely unknown, CBD has been shown to target mitochondria in other cell types, with some studies suggesting that CBD may induce mitochondrial dysfunction, oxidative stress and apoptosis [21,22,23,24]. However, the effects of CBD on trophoblast differentiation and trophoblast mitochondria are not clearly understood. Therefore, we hypothesized that CBD impairs trophoblast differentiation and alters trophoblast mitochondrial function due to its pharmacological and structural similarities with Δ^9^-THC. Hence, we sought to characterize the effects of CBD on trophoblast cell function and highlight its impacts on mitochondrial function and respiration. We report that CBD impairs trophoblast differentiation and simultaneously alters trophoblast mitochondrial function. Specifically, CBD exposure impairs key markers of CT proliferation, CT and ST differentiation and fusion, while impairing the endocrine capacity of STs. We demonstrate sublineage-specific changes to cellular and oxidative stress markers in either CTs or STs, with similar deficits to parameters of mitochondrial respiration, at the highest concentration of CBD. Finally, we illustrate that key ECS enzymes and receptors are differentially impacted by CBD treatment in CTs and STs.

## 2. Materials and Methods

### 2.1. Cell Culture

BeWo b30 cells (AddexBio, San Diego, CA, USA, C0030002) were cultured in 1X Dulbecco’s Modified Eagle’s Medium (1X DMEM containing 4.5 g/L glucose, L-glutamine and sodium pyruvate) supplemented with 10% heat-inactivated Fetal Bovine Serum (HI-FBS) (Cytiva, Marlborough, MA, USA, SH3039603) and 1% penicillin-streptomycin (Gibco, Grand Island, NY, USA, 15140122), incubated at 37 °C with 5% CO_2_. Cells were plated at a seeding density of 1 × 10^5^ cells/cm^2^ in either 12-well or 6-well plates to achieve 90% confluency, and media were replenished every 48 h. After 24 h, a subset of cells were treated with 50 ng/mL epidermal growth factor (EGF) (Gibco, Grand Island, NY, USA, PHG0311L). After 48 h, these cells were additionally treated with EGF and 50 μM forskolin (FSK) (Sigma, Burlington, MA, USA, F6886) to induce the differentiation of STs. When appropriate, the cells were treated with various concentrations of CBD immediately following the treatment with the differentiation factors (EGF and FSK). The effects of the increasing concentrations of CBD on cell proliferation and cell death were determined by MTS and LDH assays (Figure S1), respectively. The vehicle control for both CTs and STs was 0.1% methanol, and the ST vehicle control was co-treated with EGF and FSK.

### 2.2. Cell Proliferation and Viability

To determine cell proliferation, BeWo b30 cells were sub-cultured at 32,000 cells/well in a 96-well plate in 100 μL of media. Representative ST wells were initially treated with 50 ng/mL EGF after 24 h and re-treated with EGF and FSK after 48 h. Both CTs and STs were then treated with a range of CBD concentrations. Following 48 h of CBD exposure, the cells were treated with 20 μL of CellTiter 96^®^ AQ_ueous_ Non-Radioactive Cell Proliferation Assay (Promega, Madison, WI, USA, G5421) and incubated for 45 min in a humidified chamber at 37 °C with 5% CO_2_. The absorbance was measured at 490 nm using the Tecan Spark^®^ Multimode Microplate Reader (TECAN, Männedorf, Switzerland) and the readings were blank corrected. Additionally, to measure CBD-induced cytotoxicity, plasma membrane integrity was assessed using the CyQUANT^TM^ LDH Cytotoxicity Assay (Invitrogen, Waltham, MA, USA, C20300). BeWo b30 cells were cultured as described above and included two sets of untreated triplicates allocated for either “blank” or 10X lysis buffer controls. Following 48 h of CBD exposure, 50 μL of the substrate mix was added to 50 μL of media from each well for 30 min at RT after which the absorbance was measured at 490 nm and 680 nm.

### 2.3. DCFDA (2′,7′-Dichlorofluorescin Diacetate) Assay

BeWo b30 cells were seeded in a black-walled, clear-bottom 96-well microplate at 3 × 10^3^ cells/well in 100μL of media. Cells were differentiated and treated with 20 μM CBD over six days, as described above. The levels of ROS were detected using the DCFDA Cellular ROS Detection Assay Kit (Abcam, Cambridge, UK, ab113851) following the manufacturer’s protocol. The CT and ST positive controls contained 10 nM rotenone (Rot) incubated in a standard cell culture incubator with 5% CO_2_ for 4 h and 48 h, respectively. After 4 h of 10 nM rotenone treatment, all wells were treated with 5 μM DCFDA solution for 45 min and the fluorescence was quantified at an excitation/emission of 485/535 nm using the Tecan Spark^®^ Multimode Microplate Reader. The readings were normalized to total protein content using the Pierce^TM^ BCA Protein Assay Kit (ThermoFisher, Waltham, MA, USA, 23227).

### 2.4. Mitochondrial Membrane Potential

The fluorescent reagent tetraethylbenzimidazolylcarbocyanine iodide (JC-1) in the JC-1 Mitochondrial Membrane Potential Assay kit (Abcam, Cambridge, UK, ab113850) was used to measure mitochondrial membrane potential (ΔΨm) in the CBD-treated trophoblasts, according to the manufacturer’s protocol. BeWo b30 cells were plated to achieve a density of 32,000 cells/well in a black-walled, clear-bottom 96-well plate. CTs and STs were treated with 1 µM, 10 µM and 20 µM CBD or vehicle for 48 h. Following treatment, a subset of untreated CT and ST cells were washed in warm PBS (with Ca^2+^ and Mg^2+^) and incubated with 100 µM FCCP, an uncoupling agent provided as the positive control, for 4 h at 37 °C with 5% CO_2_ protected from light. After 4 h, all wells were washed with 1X dilution buffer and incubated with 20 µM JC-1 dye for 10 min at 37 °C with 5% CO_2_ protected from light. Following this incubation, the JC-1 solution was removed, the cells were washed once with 1X dilution buffer and then all wells were replaced with 1X supplemental buffer. The red fluorescence in excitation/emission (535 nm/590 nm) and green fluorescence in 475 nm/530 nm (excitation/emission) were measured using the Tecan Spark^®^ Multimode Microplate reader. The JC-1 readings were interpreted by subtracting the background readings and dividing the proportion of red fluorescence to green fluorescence in each treatment condition.

### 2.5. RNA Extraction and RT-PCR

The cells were treated in 6- or 12-well plates, harvested using 500 μL ice-cold TRIzol^TM^ reagent (ThermoFisher, Waltham, MA, USA, 15596026) and triturated to achieve homogenization, after which they were vortexed and centrifuged at 12,000× *g* for 10 min. Following this process, RNA was isolated and filtered using the Direct-zol^TM^ RNA extraction kit (Zymo, Irvine, CA, USA, R2060) according to the manufacturer’s protocol. Cells were seeded in triplicate wells and repeated six times (*N* = 18). The RNA concentrations (ng/μL) and purity values were determined using the Nanodrop^TM^ spectrophotometry instrument. Based on these measured values, 1 μg of RNA was converted to cDNA with the Applied Biosystems^TM^ High-Capacity cDNA Reverse Transcription Kit (Applied Biosystems, Waltham, MA, USA, 4368813) using the manufacturer’s protocol. RT-qPCR was performed using the Bio-Rad CFX384 Touch^TM^ (Bio-Rad, Hercules, CA, USA) Real-Time PCR Detection System for which the master mix was comprised of 5 μL GB-Amp™ Sybr Green qPCR Mix (GeneBio, Burlington, ON, Canada, P2092), primers and ddH_2_O. Analyses of the PCR results were carried out using the ΔΔCt method and normalized to two housekeeping genes (18S, β-actin). All primers used in this study are listed in Table S1.

### 2.6. Mitochondrial Respiration Assay

Cells were plated at a density of 5 × 104 cells/well in 24-well microtiter plates and the oxygen consumption rate (OCR) was measured using a Seahorse XFe24 Analyzer (Agilent, Santa Clara, CA, USA) at 37 °C. STs were differentiated with EGF and FSK according to the experimental model outlined above. Both CTs and STs were additionally treated with 1 µM, 10 µM or 20 µM of CBD. After 48 h, the culture medium was removed and replaced with an XF base medium (Seahorse Bioscience, Chicopee, MA, USA, 102365-100) supplemented with 100 mM sodium pyruvate (ThermoFisher, Waltham, MA, USA, 11360-070), 200 mM L-glutamine (ThermoFisher, Waltham, MA, USA, 25030081) and 5 mL of 45% glucose solution (Millipore-Sigma, Burlington, MA, USA, G8769), warmed to 37 °C (pH 7.4). The assay medium was pre-equilibrated at 37 °C for 1 h. The OCR was detected under basal conditions followed by the sequential injection of oligomycin (ATP-synthase inhibitor), carbonyl cyanide-4- (trifluoromethoxy)phenylhydrazone (FCCP; mitochondrial respiration uncoupler) and rotenone combined with antimycin (electron transport blockers) to final concentrations of 1, 0.3 and 0.5 μM, respectively. The OCR values were normalized to the amount of protein content from each well as determined through the BCA assay. The treatment conditions were plated in quadruplicate and repeated independently three or six times, for STs (n = 12) or CTs (n = 24), respectively.

### 2.7. Protein Extraction and Western Blot

The harvested cells were dissolved in an ice-cold radioimmunoprecipitation assay (RIPA) buffer and sonicated for three rounds of five pulses at 6–7 Hz to homogenize the samples. The total protein concentration was determined using the Pierce^TM^ BCA Protein Assay Kit (ThermoFisher, Waltham, MA, USA, 23227) along with Bovine Serum Albumin (BSA; 0–2000 μg/mL) for standardization, and subsequently measured with a 96-well plate reader at 562 nm. A total of 20–60 μg of total protein was separated on a 10–15% polyacrylamide gel and transferred to a PVDF membrane. All washes between primary and secondary antibody incubations were carried out with 1X Tris-buffered saline with 0.1% Tween^®^ 20 detergent (1X TBS-T) and the efficiency of protein transfer was visualized using the Bio-Rad ChemiDoc imaging system. Following protein transfer, the membranes were blocked in 5% BSA overnight at 4 °C and incubated with the designated primary antibody (>1:2000 dilution) for 2 h at RT. The secondary horseradish peroxidase-linked donkey anti-rabbit or sheep anti-mouse antibody (1:10,000 dilution) was incubated for 1 h at RT. Finally, we used an enzyme-linked chemiluminescence detection reagent and visualized the blot using the Bio-Rad ChemiDoc imaging system. The intensity of bands was analyzed and quantified using BioRad Version 6.1 ImageLab software (BioRad, Hercules, CA, USA) and normalized to β-actin. All the antibodies used are listed in Table S2.

### 2.8. Statistical Analyses

The statistical analyses were performed using GraphPad Prism software V6.0. Normality was assessed in all datasets in order to define the parameters. A data set with two groups was compared using a two-tailed Student’s *t*-test, whereas data sets containing two or more groups were compared with a one- or two-way analysis of variance (ANOVA) and corrected by a Tukey multiple comparison test. The data are reported as mean ± SEM. The post hoc significance will be expressed as follows: *p* < 0.05 (*), *p* < 0.01 (**), *p* < 0.001 (***).

## 3. Results

### 3.1. CBD Exposure Impairs Formation of Syncytiotrophoblasts

**Figure 1:**
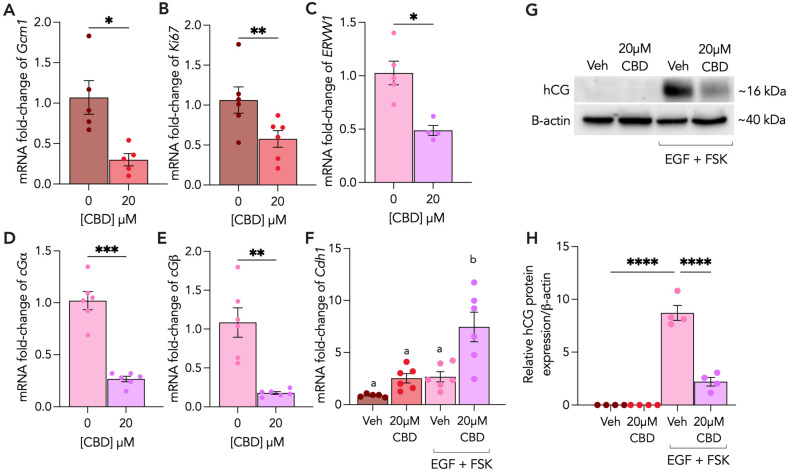
(**A**) 20 µM CBD treatment disrupts trophoblast differentiation, proliferation, fusion and hormone production. Undifferentiated (CT) and differentiated (ST) BeWo b30 cells were treated with 20 μM CBD over 48 h. (**A**,**B**) Changes in mRNA levels of Gcm1 and Ki67 were assessed in CTs and (**C**–**E**) mRNA levels of ERVW1, CGα and CGβ were assessed in STs, and all were normalized to 18S and β-Actin using RT-qPCR (n ≥ 5 biological replicates), relative to vehicle (Veh) controls. (**F**) Changes in mRNA levels of Cdh1 were assessed in both CTs and STs and were normalized to 18S and β-Actin using RT-qPCR (n ≥ 5 biological replicates). (**G**,**H**) Changes in protein levels of hCG (n = 4 biological replicates) were assessed using Western blotting and normalized to β-actin expression. Cell lysates (25 μg/lane) of each treatment were loaded on SDS-PAGE and quantified using a rabbit polyclonal anti-hCG (DAKO, GA508) antibody. CT vehicle = 0.1% methanol; ST vehicle = 0.1% methanol, EGF and FSK. Results were plotted as mean ± SEM and compared using either a Student’s *t*-test (for groups ≤ 2), or one-way ANOVA (for groups ≥ 3): *p* < 0.05 (*), *p* < 0.01 (**), *p* < 0.001 (***), *p* < 0.0001 (****). Statistically significant changes were represented by distinct letters on bar graphs where any different letter represents a significant difference of at least *p* < 0.05.

MTS and LDH assays were conducted to assess proliferation and cytotoxicity in response to various CBD concentrations. Both CTs and STs treated for 48 h with a range of CBD concentrations showed decreased proliferation and cytotoxicity between 30 and 50 μM (Figure S1A–D; *p* < 0.0001, *p* < 0.005). To determine the maximal impacts of CBD on trophoblast function, we treated CTs and STs with 20 µM CBD for 48 h and examined markers of trophoblast differentiation and fusion. First, we assessed the mRNA levels of GCM1 and Ki67, which are markers of CT differentiation and proliferation, respectively. Here, the GCM1 mRNA levels were decreased by 72% (Figure 1A) and the Ki67 levels were reduced by 50% (Figure 1B) following CBD treatment. Similarly, we showed that the transcript levels of a biochemical marker of ST differentiation, ERVW1, were decreased by 40% (Figure 1C) following 20 µM CBD treatment. To determine whether trophoblast cell fusion was impacted, we examined the transcript levels of Cdh1, or E-cadherin, a key cell adhesion protein that gradually disappears during trophoblast syncytialization [25]. Upon CBD treatment, the STs underwent a 4.7-fold increase in CDH1 (Figure 1F; *p* < 0.01). Since hCG is produced by STs in correlation with fusion, we assessed the mRNA transcript levels of hCG subunits, CGα and CGβ, as well as hCG protein expression. We observed a 0.75-fold decrease in both CGα and CGβ transcripts (Figure 1D,E) and a 74% decrease in hCG protein expression (Figure 1G,H) following CBD exposure.

### 3.2. CBD Treatment Leads to Increased Cellular and Oxidative Stress in Cytotrophoblasts

**Figure 2:**
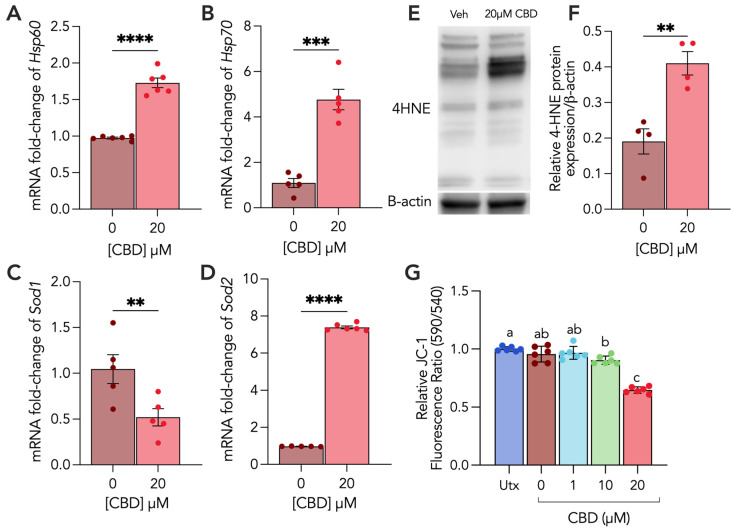
CBD treatment alters markers of mitochondrial and cellular stress and antioxidant capacity and induces oxidative stress in cytotrophoblasts. Undifferentiated (CT) BeWo b30 cells were treated with 20 μM CBD over 48 h. (**A**–**D**) Changes in mRNA levels of HSP60, HSP70, SOD1 and SOD2 were normalized to 18S and β-Actin and were assessed using RT-qPCR (n ≥ 5 biological replicates), relative to vehicle control (Veh). (**E**,**F**) Changes in protein levels of 4HNE (n = 4 biological replicates) were assessed using Western blotting and normalized to β-actin expression. Cell lysates (25 μg/lane) of each treatment were loaded on SDS-PAGE and probed using a rabbit polyclonal anti-4HNE (Abcam, ab46545) antibody. (**G**) Mitochondrial membrane potential (ΔΨm) was determined in both untreated (Utx) cells and following 0 (vehicle), 1, 10 and 20 µM of CBD treatment (n = 6 biological replicates per treatment condition) in CTs using the JC-1 assay kit (Abcam, ab113850). CT vehicle = 0.1% methanol. Results were plotted as mean ± SEM and compared using either Student’s *t*-test (for groups ≤ 2), or one-way ANOVA (for groups ≥ 3): *p* < 0.01 (**), *p* < 0.001 (***), *p* < 0.0001 (****). Statistically significant changes were represented by distinct letters on bar graphs where any different letter represents a significant difference of at least *p* < 0.05.

To understand the biochemical changes underlying the effects of CBD on CTs, we first sought to characterize two markers of cellular stress, HSP60 and HSP70, as well as two key antioxidant markers, superoxide dismutases SOD1 and SOD2, following CBD exposure. We observed a 30% decrease and 3.7-fold increase in HSP60 and HSP70 mRNA levels, respectively (Figure 2A,B). We found that SOD2 mRNA levels were increased by 6.4-fold (Figure 2D), whereas SOD1 levels were decreased 0.5-fold (Figure 2C). Given the considerable reduction in the SOD1 antioxidant defense component, we further assessed intracellular ROS levels by performing the DCFDA assay in CBD-treated CTs and found that intracellular ROS levels remained unchanged with CBD treatment (Figure S2). Considering the changes to antioxidant defenses, we assessed the cellular levels of 4HNE, a marker of lipid peroxidation and oxidative damage [26]. CBD treatment resulted in a 53% increase in 4HNE protein adducts (Figure 2E,F). Finally, we assessed mitochondrial membrane potential (ΔΨm) and observed a 10% decrease in mitochondrial membrane potential with 10 µM CBD and a 36% decrease with 20 µM CBD (Figure 2G).

### 3.3. CBD Treatment Leads to Increased Mitochondrial and Oxidative Stress in Syncytiotrophoblasts

**Figure 3:**
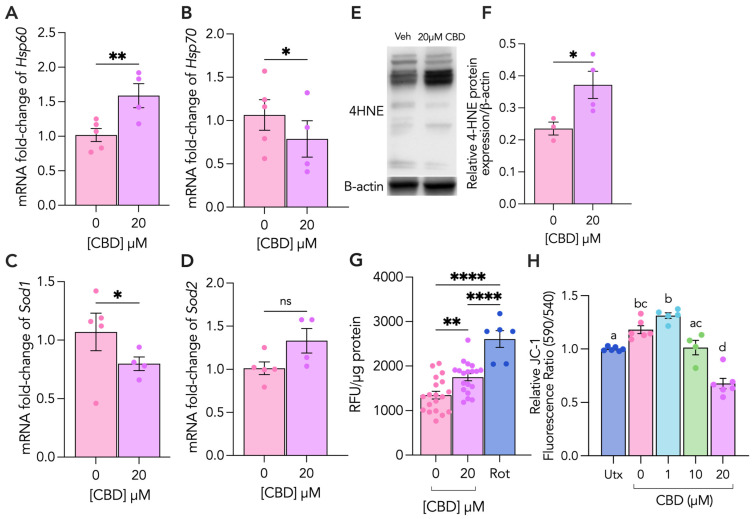
CBD treatment alters markers of mitochondrial and cellular stress, antioxidant capacity and differentiation, and induces oxidative stress in syncytiotrophoblasts. Differentiated (ST) BeWo b30 cells were treated with 20 μM CBD over 48 h. (**A**–**D**) Changes in mRNA levels of HSP60, HSP70, SOD1 and SOD2 were normalized to 18S and β-Actin and were assessed using RT-qPCR (*N* ≥ 5 biological replicates), relative to vehicle control (Veh). (**E**,**F**) Changes in protein levels of 4HNE (n = 4 biological replicates) were assessed using Western blotting and normalized to β-actin expression. Cell lysates (25 μg/lane) of each treatment were loaded on SDS-PAGE and probed using a rabbit polyclonal anti-4HNE (Abcam, ab46545) antibody. (**G**) Intracellular ROS levels were quantified using the DCFDA assay (Abcam, ab113851) in ST cells following treatment with 20 μM CBD compared to vehicle control (n = 24 biological replicates). A total of 10 nM of rotenone (Rot) was used as a positive control and results were normalized to total protein content determined through the BCA assay. (**H**) Mitochondrial membrane potential (ΔΨm) was determined in both untreated (Utx) cells and following 0 (vehicle), 1, 10 and 20 µM of CBD treatment (n = 6 biological replicates per treatment condition) in STs using the JC-1 assay kit (Abcam, ab113850). ST vehicle = 0.1% methanol, EGF, FSK. Results were plotted as mean ± SEM and compared using either Student’s *t*-test (for groups ≤ 2), or one-way ANOVA (for groups ≥ 3): *p* < 0.05 (*), *p* < 0.01 (**), *p* < 0.0001 (****). Statistically significant changes were represented by distinct letters on bar graphs where any different letter represents a significant difference of at least *p* < 0.05.

To provide further insights into the CBD-induced changes to ST metabolism and cellular and oxidative stress, HSP60, HSP70, SOD1 and SOD2 mRNA levels were characterized following CBD treatment. We observed a 56% increase in HSP60 (Figure 3A) transcript levels and a 25% decrease in both HSP70 and SOD1 mRNA levels (Figure 3B,C). We sought to investigate whether this CBD-induced mitochondrial stress was associated with changes in intracellular ROS levels by conducting a DCFDA assay. Our results showed a 23% increase in intracellular ROS production following CBD treatment (Figure 3G). Consistent with this observation, we performed immunoblotting for 4HNE and showed a 37% increase in 4HNE protein adducts (Figure 3H).

### 3.4. 20 µM CBD Reduces Oxygen Consumption Rate, Basal Respiration and ATP-Linked Respiration in Both Cytotrophoblasts and Syncytiotrophoblasts

**Figure 4:**
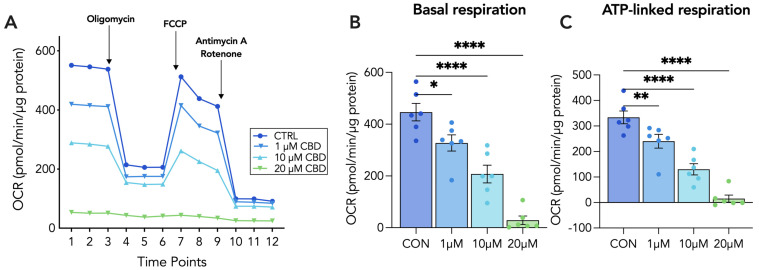
Mitochondrial function is impaired following 20 µM CBD treatment in CT cells. Undifferentiated (CT) BeWo b30 cells were treated with 1, 5, 10 and 20 µM CBD for 48 h at which point the mitochondrial stress test was conducted. Basal OCR measurements were obtained using the Seahorse XFe24 Analyzer and following the sequential addition of compounds oligomycin, FCCP and antimycin A/rotenone, with values being normalized to total protein content via BCA assay (μg/mL). The detection of OCR was performed with four biological replicates per experiment, for each treatment condition, and repeated at least four more times. Group mean and SEM (**A**–**C**) are displayed. Arrows indicate addition of the respective compounds. Significance was assessed by Student’s *t*-test (* *p* < 0.05, ** *p* < 0.01, **** *p* < 0.0001). (**A**) Representative oxygen consumption rate (OCR) tracing, (**B**) basal respiration, and (**C**) ATP-linked respiration.

**Figure 5:**
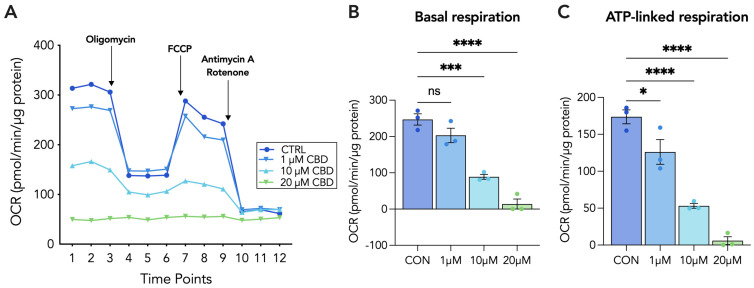
Mitochondrial function is impaired following 20 µM CBD treatment in STs. Differentiated (ST) BeWo b30 cells were treated with 1, 5, 10 and 20 µM CBD for 48 h at which point the mitochondrial stress test was conducted. Basal OCR measurements were obtained using the Seahorse XFe24 Analyzer and following the sequential addition of compounds oligomycin, FCCP and antimycin A/rotenone, with values being normalized to total protein content via BCA assay (μg/mL). Detection of OCR was performed with four biological replicates per experiment, for each treatment condition, and repeated four more times. Group mean and SEM (**A**–**C**) are displayed. Arrows indicate addition of respective compounds. Significance was assessed by Student’s *t*-test (* *p* < 0.05, *** *p* < 0.001, **** *p* < 0.0001; ns, non-significant). (**A**) Representative oxygen consumption rate (OCR) tracing, (**B**) basal respiration and (**C**) ATP-linked respiration.

To further establish the impacts of CBD on parameters of mitochondrial respiration, we conducted the Agilent Seahorse XF Mito Stress Test assay to measure the mitochondrial oxygen consumption rate (OCR). For both CTs and STs, cells were treated with a range of concentrations of CBD, including 1 µM, 10 µM and 20 µM for 48 h prior to the sequential addition of oligomycin, FCCP and antimycin A/rotenone. We determined that basal respiration in CTs was dose-dependently decreased, with 1 µM and 20 µM of CBD significantly reducing basal respiration by 26% and 93%, respectively (Figure 4B). In STs, we observed significant reductions in basal respiration induced by the 10 µM and 20 µM concentrations of CBD (Figure 5B). Moreover, CBD dose-dependently decreased ATP-linked respiration in both CTs and STs. In both cell types, 1 µM and 20 µM of CBD significantly decreased ATP-linked respiration by approximately 27% and 95%, respectively (Figure 4C and Figure 5C).

### 3.5. Endocannabinoid Receptors and Enzymes Are Differentially Altered by CBD Treatment in Cytotrophoblasts and Syncytiotrophoblasts

**Figure 6:**
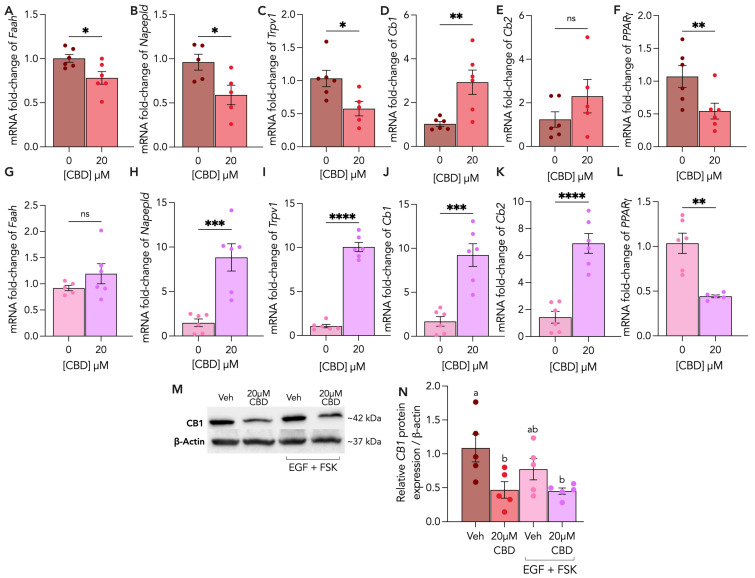
Endocannabinoid enzymes and receptors are differentially altered in CTs and STs following CBD treatment. Undifferentiated (CT) and differentiated (ST) BeWo b30 cells were treated with 20 μM CBD over 48 h. (**A**–**F**) Changes in mRNA levels of FAAH, NAPE-PLD, TRPv1, CB1, CB2 and PPARγ in CTs were normalized to 18S and β-Actin and were assessed using RT-qPCR (n ≥ 5 biological replicates), compared to CT vehicle control. (**G**–**L**) Changes in mRNA levels of FAAH, NAPE-PLD, TRPv1, CB1, CB2 and PPARγ in STs were normalized to 18S and β-Actin and were assessed using RT-qPCR (n ≥ 5 biological replicates), compared to ST vehicle control. (**M**,**N**) Changes in protein levels of CB1 (n = 5 biological replicates) were assessed using Western blotting and normalized to β-actin expression. Cell lysates for CB1 (60 μg/lane) of each treatment were loaded on SDS-PAGE and quantified using a polyclonal rabbit anti-CB1 (Cayman) antibody. CT vehicle = 0.1% methanol; ST vehicle = 0.1% methanol, EGF, FSK. Results were plotted as mean ± SEM and compared using either Student’s *t*-test (for groups ≤ 2), or one-way ANOVA (for groups ≥ 3): *p* < 0.05 (*), *p* < 0.01 (**), *p* < 0.001 (***), *p* < 0.0001 (****). Statistically significant changes were represented by distinct letters on bar graphs where any different letter represents a significant difference of at least *p* < 0.05.

To characterize the impacts of CBD on ECS components, we assessed the transcriptional levels of key endocannabinoid enzymes and receptors, including FAAH, NAPE-PLD, TRPV1, CB1, CB2 and PPARγ, using RT-qPCR, and further quantified the protein expression of CB1 using immunoblotting. We observed that, following CBD treatment for 48 h, the CTs had significantly downregulated FAAH (0.25-fold), NAPE-PLD (0.5-fold), TRPV1 (0.5-fold) and PPARγ (0.5-fold) gene expression (Figure 6A–C,F), whereas CB1 mRNA levels were upregulated by 64% relative to untreated control (Figure 6D). In contrast, NAPE-PLD and TRPV1 mRNA levels were increased by 7.4-fold (Figure 6H) and 9-fold (Figure 6I) in STs, respectively, following CBD treatment. Additionally, we demonstrated that CBD treatment increased CB1 and CB2 mRNA levels by 7.5-fold (Figure 6J) and 5.5-fold (Figure 6K), respectively, and decreased PPARγ mRNA levels by 0.5-fold (Figure 6L) in STs. Furthermore, we observed a significant downregulation of CB1 (Figure 6M,N) protein expression in the CBD-treated CTs and no significant changes in STs.

## 4. Discussion

Stem cell differentiation is a crucial process in development and regeneration that involves mitochondrial signaling. Emerging evidence supports the hypothesis that mitochondria mediate stem cell fate decisions through the production of metabolic byproducts that influence self-renewal and commitment cues in several tissue types [27,28], such as with neural stem cells (NSCs) [29] and hematopoietic stem cells (HSCs) [30]. Villous trophoblast differentiation, the transition from cytotrophoblasts to syncytiotrophoblast, is necessary for proper placentation. Indeed, trophoblast mitochondria undergo distinct morphological and bioenergetic shifts that accompany these specific differentiation states, suggesting inherent metabolic differences between cytotrophoblasts and syncytiotrophoblasts [31,32,33], which may necessitate the need for mitochondrial adaptations during syncytialization [28]. Our previous work highlighted that Δ^9^-THC is capable of disrupting trophoblast syncytialization as well as mitochondrial function and dynamics [19]; however, the impacts of CBD on trophoblast differentiation and metabolism remain elusive. In our present study, we sought to address the hypothesis that CBD impairs trophoblast mitochondrial function and consequently hinders trophoblast differentiation and fusion. To investigate this question, we employed the BeWo b30 cell line, which is a trophoblastic cell line able to undergo fusion and syncytialization following cAMP stimulation (following forskolin treatment) and which also satisfies the defining criteria of trophoblasts [34,35]. To date, this cell line is the most appropriate in vitro model of villous trophoblast differentiation, apart from primary trophoblast culture, and allows us to investigate functional outcomes, such as syncytial fusion and endocrine function [36,37,38,39].

One of the key functions of the syncytium is to produce essential pregnancy hormones, including human chorionic gonadotropin (hCG), human placental lactogen (hPL), progesterone and insulin-derived growth factor 2 (IGF2), that are involved in pregnancy maintenance and/or fetal development [40]. hCG is an important hormone indicator of trophoblast differentiation and pregnancy success [41]. Apart from its function in promoting progesterone production in early pregnancy, hCG produced by differentiated STs also promotes the fusion of proliferating CTs by stimulating adenylyl cyclase to increase levels of cAMP [42,43]. Le Vee et al. (2014) reported that polycyclic aromatic hydrocarbons (PAHs) are capable of triggering trophoblast syncytialization by upregulating βhCG as well as other biochemical markers of syncytialization [44]. We found that biochemical markers of trophoblast differentiation, GCM1 and ERVW1, and a marker of cell proliferation, Ki67, were all downregulated with 20 µM CBD treatment. Using hCG as an indicator of the extent of fusion, we show that hCG mRNA and protein levels were reduced following CBD treatment. In BeWo cells, similar reductions in hCG and E-cadherin resulted in lower cell-to-cell fusion following Δ^9^-THC treatment [19], suggesting that CBD may be impairing cellular fusion in our model. hCG has also been established as a pro-angiogenic factor [45,46] as it increases capillary formation in a human 3D in vitro model of angiogenesis and induces neovascularization in a chicken chorioallantoic membrane (CAM) model [47]. In human villous tissue from missed abortions, decreased serum hCG and VEGF levels were associated with downregulated VEGF-MEK/ERK signaling pathway components, suggesting the dysregulation of villous angiogenesis [48]. In mice, hCG was shown to stimulate higher serum levels of soluble VEGFR1 and free VEGFA [49]. Ultimately, decreased hCG levels may result in defects in placental angiogenesis and vasculogenesis, as well as consequences to fetal organ growth and development [50]. Although the link between cannabinoid exposure and hCG regulation has not been thoroughly established in vivo, one study demonstrated that gestational Δ^9^-THC exposure resulted in dysregulated placental vasculature in rats, suggesting the possibility for cannabinoid-induced dysregulation of angiogenic factors during placentation [51]. Moreover, as E-cadherin is a prominent cell-to-cell fusion protein whose expression indicates the presence of cell boundaries, its expression decreases as cell boundaries merge during syncytialization [52]. Interestingly, the transcript levels of CDH1 were significantly upregulated following CBD treatment in STs, supporting the view that exposure to CBD leads to reduced cell–cell fusion. Likewise, we have previously demonstrated the anti-fusogenic properties of Δ^9^-THC in BeWo cells through decreased CGα and CGβ mRNA levels, as well as the decreased secretion of hCG following Δ^9^-THC treatment [19], suggesting that CBD and Δ9-THC may act through similar mechanisms to induce their effects. In fact, Δ^9^-THC and CBD are able to interact with CB1 as partial or weak agonists, respectively, and CB1 activation is coupled with Gi/o proteins that inhibit cAMP formation [53]. Considering that FSK-induced cAMP stimulation is necessary to promote differentiation [54] and syncytialization [55,56], we postulate that cannabinoids could be interfering with the action of cAMP-dependent pathways regulating these critical processes involved with the formation of the maternal–fetal interface.

As mitochondrial function and respiration are central to stem cell fate decisions, through downstream redox signaling [57,58], it is important to understand how CBD activity may impact trophoblast mitochondria, especially considering the increasing evidence alluding to the impacts of Δ^9^-THC on mitochondria [19,59]. Aside from NADPH oxidases (NOXs), functional and active mitochondria contribute to reactive oxygen species (ROS) levels due to electron leakage from the mitochondrial respiratory chain [60,61]. Oxidative stress occurs when the capacity of cellular antioxidant defense mechanisms is overwhelmed by ROS production. SOD1 and SOD2 are key antioxidants primarily situated in the cytoplasm and mitochondrial intermembrane space (IMS) or mitochondrial matrix, respectively, and which catalyze the conversion of superoxides into molecular oxygen and hydrogen peroxide [62,63,64]. There is debate in the literature as to the definitive role of CBD in redox modulation, with studies attributing CBD as having either pro-oxidant [22] or anti-oxidant effects [65,66]; however, these paradoxical functions of CBD could be influenced by the existing intracellular redox state as well as the concentrations of CBD employed. In our study, we observed significant downregulations in SOD1 mRNA in both CTs and STs, whereas SOD2 expression was upregulated in CTs, following 20 µM CBD treatment. In addition, we observed that HSP60, a mitochondrial chaperone, was upregulated in both CTs and STs; however, the cellular stress marker, HSP70, was dramatically upregulated only in CTs. These results suggest that CBD exposure induces mitochondrial and cellular stress to a greater extent in CTs as compared to STs, likely due to their greater reliance on mitochondrial function and higher metabolic activity to support their proliferative function. Previously, our group has demonstrated that 20 µM Δ^9^-THC treatment in BeWo cells resulted in the significant upregulation of HSP60, HSP70, SOD1 and SOD2 mRNA, suggesting that CBD may be acting via different antioxidant response pathway(s) as compared to Δ^9^-THC. In contrast to observations in the STs, the ROS levels in CTs remained unchanged with CBD exposure, possibly due to the drastic upregulation of SOD2, which could speak to the source of ROS production being primarily mitochondrial. Importantly, mitochondrial ROS production in differentiated STs has been shown to impair key trophoblast functions, such as hormone production and cellular fusion, while altering mitochondrial morphology [67]. Therefore, this apparent CBD-induced ROS production may explain the impairments to ST fusion, differentiation and hCG production in STs. We found that a biomarker of lipid peroxidation and oxidative stress, 4HNE [68], was significantly upregulated in both sub-lineages, indicating the presence of CBD-induced oxidative stress. The different impacts of CBD on ROS production in CTs and STs may depend on their baseline metabolic activity and levels of ROS, as the literature suggests that CBD could possess pro-oxidant and anti-oxidant functions depending on the intracellular niche. There exists a direct link between ROS production, oxidative damage and the disruption of mitochondrial membrane potential, as the peroxidation of mitochondrial phospholipids in the inner or outer mitochondrial membranes leaves the mitochondrial barrier permeable to protons, ultimately dissipating the proton gradient [69,70]. More specifically, when 4HNE forms adducts with mitochondrial uncoupling proteins (UCPs) and adenine nucleotide translocases (ANTs), this leads to an increased proton leakage and partial mitochondrial uncoupling [71,72]. We found that 20 µM CBD decreased mitochondrial membrane potential in both CTs and STs, which is consistent with the previous literature demonstrating that oxidative stress is coupled with disrupted mitochondrial membrane potential in high-fat diet (HFD)-fed mice [73]. Ultimately, these results support the hypothesis that CBD disrupts mitochondrial function, by dysregulating redox homeostasis and leading to oxidative stress.

Mitochondrial bioenergetics play an important role in cellular differentiation cues. The dynamic ability of mitochondria to accommodate fluctuating cellular energetic demands, by favoring either oxidative phosphorylation (OXPHOS) or glycolysis for ATP production, leads to the production of distinct metabolic byproducts that participate in downstream signaling. CBD has been shown to decrease ATP production and basal OCR in both neuroblastoma cells and isolated rat brain mitochondria at concentrations >3.75 µM [74]. Additional studies have demonstrated that cannabinoids can alter and inhibit electron transport chain (ETC) complex subunits as well as reduce respiration [19,75,76,77]. In THP monocytes, a higher micromolar concentration of CBD diminished both maximal respiration and ATP production by 58% and 60%, respectively [78]. Considering that the existing literature supports a potential concentration-dependent or biphasic effect of cannabinoids on mitochondrial respiration, we included a concentration range from 1–20 µM CBD in our mitochondrial respiration assay. Our results revealed a dose-dependent decrease in basal respiration and ATP production in both CTs and STs, which is consistent with the aforementioned findings. Consistent with the literature, our findings showed that untreated CTs were twice as metabolically active as STs based on basal respiration measurements. Impairments to basal respiration and ATP production were significant even at the lowest concentrations of CBD, suggesting that trophoblast mitochondria are sensitive to physiologically relevant concentrations of CBD. In contrast, no changes were observed in the basal respiration of STs following 20 µM Δ^9^-THC treatment, whereas ATP production was partially diminished [19]. Recent studies suggest that CBD has an affinity for the mitochondrial voltage-dependent anion channel 1 (VDAC1), where it has been demonstrated to decrease channel conductance [23]. In addition, Mahmoud and colleagues have described a mechanism wherein CBD increases glycolytic capacity and inhibits OXPHOS in HRPC cells by modulating VDAC1 and hexokinase II (HKII) coupling [79], which is consistent with our findings that OXPHOS is impaired following CBD treatment in trophoblasts. Collectively, the differences in the magnitude of the mitochondrial respiration impairments observed between CBD and Δ^9^-THC may be explained by their pharmacological actions on other mitochondrial proteins, such as VDAC1. Overall, the transient metabolic shift from glycolysis to OXPHOS is a highly regulated signal for stem cell commitment and differentiation, and further studies should be aimed at characterizing this metabolic switch in trophoblasts, by investigating the glycolytic capacity of trophoblasts following CBD treatment [31].

Cannabinoid pharmacology is complex and has been increasingly shown to involve both canonical and non-canonical receptors [80]. Considering that trophoblast differentiation and function are contingent on the interplay of ECS components [81,82], we chose to characterize the impacts of CBD on the gene expression levels of the ECS receptors CB1, CB2, TRPV1 and PPARγ, as well as the key ECS enzymes FAAH and NAPE-PLD, in CTs and STs. Importantly, we found differential impacts on gene expression markers between CTs and STs following CBD treatment, suggesting that the effects of CBD may be altered by differentiation status. The transcript levels of FAAH, NAPE-PLD and TRPV1 were significantly decreased in CTs, whereas NAPE-PLD and TRPV1 were observed to be increasing in STs following 20 µM CBD exposure. Although CB1 transcripts were significantly upregulated in both CTs and STs, CB2 expression was only upregulated in STs. Despite CBD acting as a negative allosteric modulator of CB1 and an antagonist of CB2 [83], some studies have shown that CBD may indirectly increase levels of AEA, an endogenous agonist of CB1/2, by preventing AEA hydrolysis via weak FAAH inhibition [84,85]. Considering that NAPE-PLD and FAAH synthesize and degrade AEA, respectively, along with the observation that NAPE-PLD is profoundly increased following CBD treatment in STs, it is possible that AEA levels may be increased with CBD treatment. This is consistent with reports suggesting that AEA may attenuate trophoblast fusion [27,86] as well as those demonstrating that CBD and Δ^9^-THC inhibit the fatty-acid binding proteins (FABPs) involved in shuttling AEA to FAAH for breakdown [87]. Moreover, in particular brain regions of wild-type (WT) mice, CBD increased the activity of NAPE-PLD and therefore increased levels of AEA, effects which were not observed in NAPE-PLD knockout mice [88]. Furthermore, a randomized, double-blind clinical trial conducted by Leweke and colleagues found significant increases in the serum AEA levels of schizophrenic patients following the administration of CBD [89].

In addition to the regulatory enzymes for endocannabinoid homeostasis, we also noted that CBD impacts key receptors that are targeted by endocannabinoids. We previously demonstrated that Δ^9^-THC, a partial agonist of both CB1 and CB2, impaired BeWo cell fusion through CB1 and CB2 agonism [19]. Interestingly, while the mRNA levels of CB1 were upregulated in both sub-lineages following CBD treatment, CB1 protein expression was significantly decreased in CTs. It has been suggested that decreased CB1 protein expression is reflective of CB1 desensitization and downregulation caused by persistent CB1 activation [90]. Therefore, if CBD indirectly activates CB1 and causes its downregulation at the protein level, it is possible that the effects of CBD result in a compensatory upregulation of the expression of CB1 and CB2. In both CTs and STs, we observed decreases in PPARγ mRNA levels following CBD treatment. To date, no intracellular mechanism has been identified linking canonical cannabinoid receptor activation to the regulation of cellular antioxidants; however, the nuclear receptor PPARγ contains PPAR response elements in several antioxidant promoter regions, including SOD1 and SOD2 [91]. Both CBD and Δ^9^-THC have been established as agonists of PPARγ [92,93,94] and are capable of increasing its transcriptional activity, which could presumably lead to the increased transcription of the antioxidants SOD1 and SOD2 downstream. Interestingly, we observed that CBD treatment downregulated PPARγ transcripts in both CTs and STs, which may explain the CBD-induced downregulation of SOD1 in CTs and STs. Indeed, as PPARγ has been shown to promote trophoblast differentiation [95], the ability of CBD to downregulate PPARγ transcript levels could further suggest impaired trophoblast differentiation.

Altogether, our findings demonstrate the capacity of CBD to impair trophoblast differentiation and fusion, while concomitantly impairing mitochondrial respiration and inducing oxidative stress, as well as dysregulating antioxidant expression and ECS components. Considering the variability that exists in the mode, amount and duration of cannabis consumption, the physiological concentrations of CBD are reported to range between 5 nM and 334 nM [96,97,98,99]. As the pharmacodynamics of CBD are contingent on its lipophilic nature, it is possible for CBD to accumulate in fatty tissues at a lower micromolar range. The concentrations employed in our study were higher than those reported in clinical studies, which is a limitation of this work. However, our findings support the emerging hypothesis that cannabinoids, such as CBD and Δ9-THC, are capable of disrupting cellular differentiation by dysregulating mitochondrial bioenergetics [27]. Taken more broadly, the fine-tuned regulation of mitochondrial bioenergetics is required for proper tissue regeneration and organ development in other tissues, which makes it crucial to understand the consequences of prenatal and postnatal cannabinoid exposure on stem cell regulation. The importance of understanding the consequences of perinatal exposure to cannabinoids is highlighted in observations that demonstrate that defective angiogenesis and decreased hCG production have been associated with an increased risk of pre-eclampsia (PE) [100] and miscarriage [101]. Natale et al. (2020) demonstrated that Δ9-THC exposure in utero compromised fetal growth and altered placental development in a rat model of pregnancy, suggesting the detrimental impacts of Δ9-THC [51]. Importantly, gestational CBD exposure was associated with postnatal glucose intolerance in rat offspring, indicating the long-term consequences of gestational cannabis use [102].

## Data Availability

The original contributions presented in this study are included in the article/supplementary material, further inquiries can be directed to the corresponding author/s.

## Supplementary Materials

The following supporting information can be downloaded at: https://www.mdpi.com/article/10.3390/cells13060486/s1, Figure S1: CBD negatively impacts BeWo-b30 cells plasma membrane integrity and viability at high doses; Figure S2: 20 µM CBD treatment does not alter ROS levels in CTs. Table S1: A list of human primer sequences used for RT-qPCR; Table S2: A list of antibodies used for immunoblotting.
