# Supplementary material for: Cannabidiol Disrupts Mitochondrial Respiration and Metabolism and Dysregulates Trophoblast Cell Differentiation
Source: Cells. 2024 Mar 11;13(6):486. doi: 10.3390/cells13060486 (PMC10968792; doi:10.3390/cells13060486)
Supplement: Supplementary file 1 [file cells-13-00486-s001.zip › cells-2862091-supplementary.pdf]

cells

MDPI

Supplemental Data

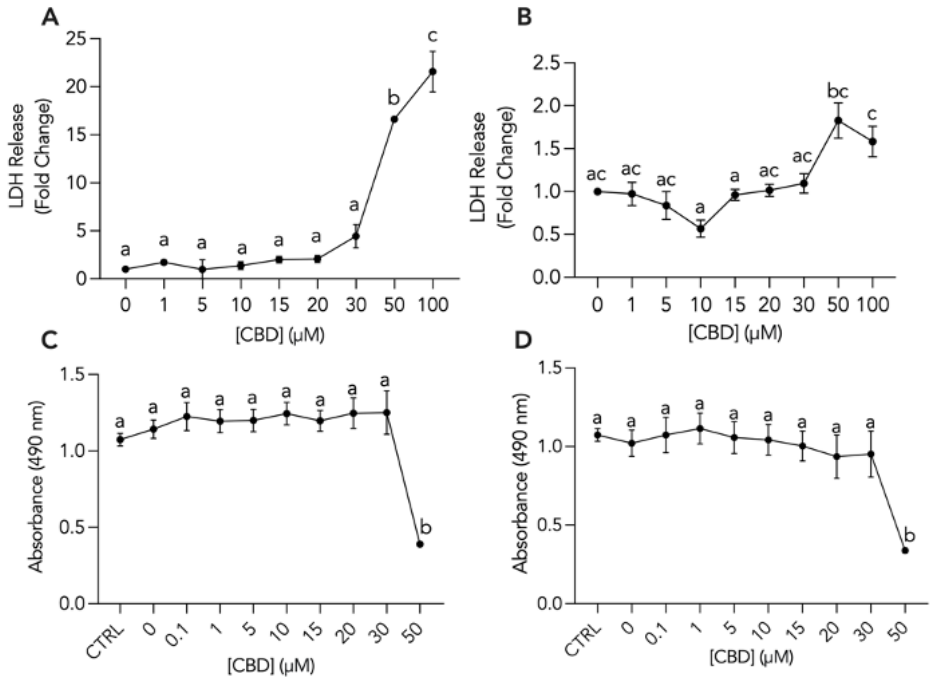

Figure S1. CBD negatively impacts BeWo-b30 cells plasma membrane integrity and viability at high doses. Undifferentiated (CT) and differentiated (ST) BeWo-b30 cells were treated with CBD at varying concentrations and subjected to both (**A**,**B**) LDH (n = 36 biological replicates) and (**C**,**D**) MTS (n = 24 biological replicates) assays. LDH release in (**A**) CTs and (**B**) STs treated with CBD. MTS absorbance readings in (**C**) CTs and (**D**) STs measured at 490nm and 680nm. Results were plotted as mean ± SEM and compared using a one-way ANOVA. Statistically significant changes were represented by distinct letters on bar graphs.

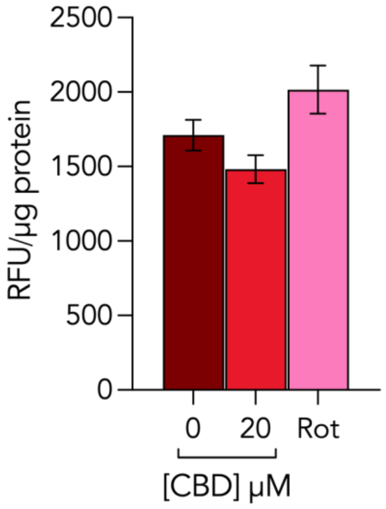

Figure S2. 20μM CBD treatment does not alter ROS levels in CTs. Undifferentiated (CT) BeWo b30 cells were treated with 20μM CBD over 48 hours. Intracellular ROS levels were quantified using the DCFDA assay in CT cells following treatment with 20 μM CBD compared to vehicle control (0.1% methanol). 10 nM of rotenone was used as a positive control and results were normalized to total protein content determined through the BCA assay. Significant differences were determined by a one-way ANOVA.

Table S1. A list of human primer sequences used for RT-qPCR.

Gene	Forward (5'→3')	Reverse (3'→5')
β-actin	TTACAGGAAGTCCCTTGCCATC	GCAATGCTATCACCTCCCCTG
18S	CACGCCACAAGATCCCA	AAGTGACGCAGCCCTCTATG
GCM1	CCTCTGAAGCTCATCCCTTGC	ATCATGCTCTCCCTTTGACTGG
ERVW-1	GTTAATGACATCAAAGGCACCC	CCCCATCTCAACAGGAAAACC
Ki67	CGTCCCAGTGGAAGAGTTGT	CGACCCCGCTCCTTTTGATA
HSP60	GAAGGCATGAAGTTTGATCG	TTCAAGAGCAGGTACAATGG
HSP70	GGAGTTCAAGAGAAAACACAAG	AAGTCGATGCCCTCAAAC
SOD1	AAAGATGGTGTGGCCGATGT	CAAGCCAAACGACTTCCAGC
SOD2	GCTCCGGTTTTGGGGTATCT	GATCTGCGCGTTGATGTGAG
TRPV1	CAGCAGCGAGACCCCTAA	CCTGCAGGAGTCGGTTCA
FAAH	TGAGCCTGAATGAAGGGGTG	ACTTCCATGGGTTCACGGTC
NAPE-PLD	GAGTCACATGGCTGGGACAT	GGACCGCATCTATTGGAGGG
CGα	GCAGGATTGCCCAGAATGC	TCTTGGACCTTAGTGGAGTGG
CGβ	ACCCCTTGACCTGTGAT	CTTTATTGTGGGAGGATCGG
CB1	ATAGCCATTGTGATCGCCGT	CCCCGATCCAGAACATCAGG
CB2	ACTCAACAGGTGCTCTGAGTG	AGGCTTTGGGTTGTGTTGTG
Cdh1	GTGCCTGAGAACGAGGCTAA	TGCATCTTGCCAGGTCCTT
PPARγ	AGTGGGGATGTCTCATAATGCC	AGCTCAGCGGACTCTGGATTC

Table S2. A list of antibodies used for immunoblotting.

Antibody	Manufacturer, catalog #	Species raised in	1° dilution used
CB1	Cayman, 10006590	Rabbit polyclonal	1:5000
4HNE	Abcam, ab46545	Rabbit polyclonal	1:1000
hCG	DAKO, GA508	Rabbit polyclonal	1:1000
